# Supplementary material for: Modular, inducible, and titratable expression systems for Escherichia coli and Acinetobacter baumannii
Source: Microbiol Spectr. 2024 Sep 20;12(11):e01306-24. doi: 10.1128/spectrum.01306-24 (PMC11536989; doi:10.1128/spectrum.01306-24)
Supplement: Supplemental figures — Fig. S1 to S5. [file spectrum.01306-24-s0001.pdf]

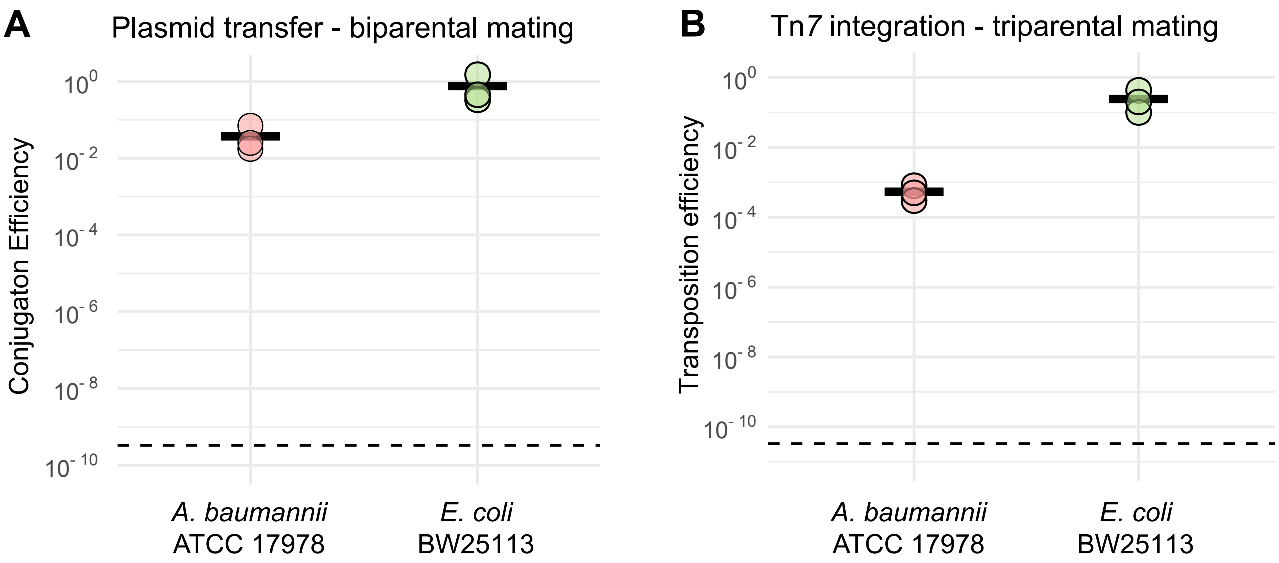

C

Co-integrates: CarbR Tn*7* transconjugants

	Strain	
	A. baumannii ATCC 17978	*E. coli* BW25113
Tn*7* vector - empty	2/40	0/40
Tn*7* vector - sfGFP	3/40	1/40

Figure S1. Conjugation and transposition efficiencies.

(A) Dot plot of conjugation efficiency of the replicative vector to the recipient strain (*A. baumannii* ATCC 17978 or *E. coli* BW25113) after ~3-hour incubations. Bars represent mean efficiencies (n=3). Dashed line is drawn at the limit of detection, defined by the maximum number of colony-forming units (CFUs) observed on non-selective plates in each experiment. **(B)** Dot plot showing transposition efficiency of the Tn*7* transposon into the att_{Tn7} site of *A. baumannii* ATCC 17978 or *E. coli* BW25113 after ~4-hour incubation with donor strains, one carrying the Tn*7* transposon vector and another carrying the Tn*7* transposase vector. Bars represent mean efficiencies (n=3); dashed line shows limit of detection. **(C)** Table showing instances of carbenicillin-resistant (CarbR) transconjugants of 40 isolates tested for Tn*7* co-integrates in *A. baumannii* and *E. coli*.

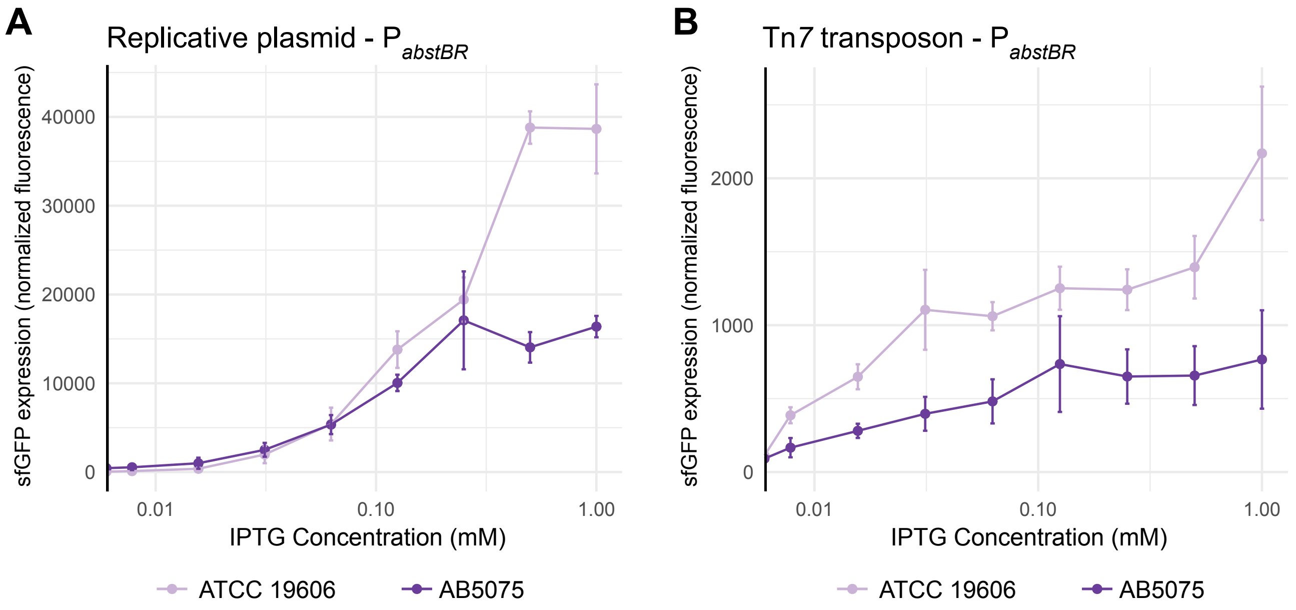

Figure S2. Titration of P_{abstBR} expression in *A. baumannii* strains.

Expression from **(A)** the replicative plasmid vector or **(B)** the Tn*7* transposon. Plots shown are normalized sfGFP levels expressed from P_{abstBR} across IPTG concentrations for *A. baumannii* ATCC 19606 and *A. baumannii* AB5075. Error bars represent standard deviation (n=3 biological replicates for replicative vector, n=6 biological replicates for Tn*7* transposon).

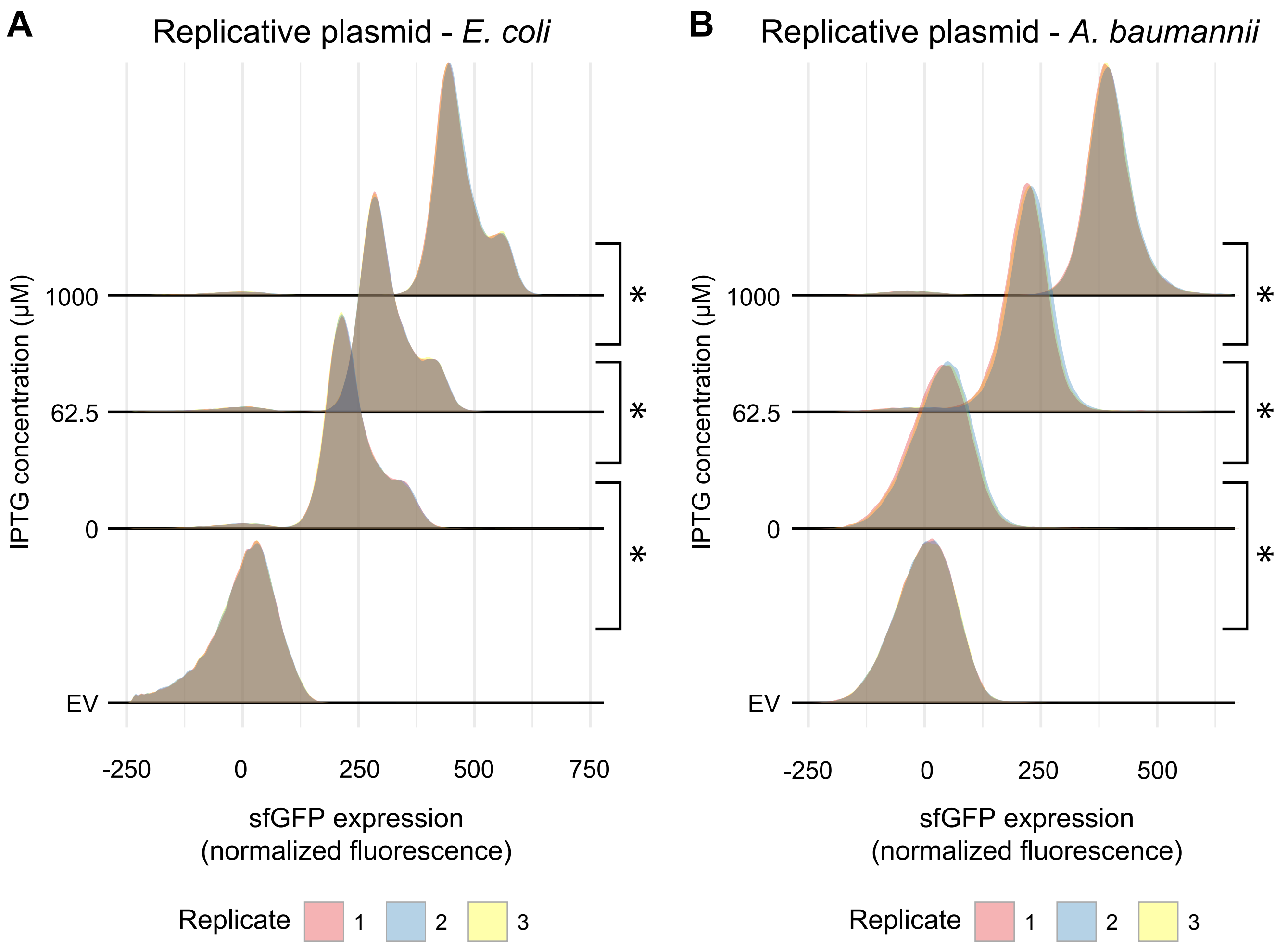

Figure S3. Titration of P$_{abstBR}$ expression at the single-cell level across replicates. Titration of sfGFP expression from the replicative expression vector, under control of P$_{abstBR}$, in **(A)** *E. coli* BW25113 or **(B)** *A. baumannii* ATCC 17978 at mid-log growth phase. Ridgeline plots depict density plots of sfGFP fluorescence for cells induced at 0, 62.5, or 1000 µM IPTG, measured by flow cytometry (~95,000 cells per sample). EV are empty vector (no GFP) control samples in 1 mM IPTG. 3 biological replicates for each sample are overlaid. Asterisks indicate significant differences between samples (Welch's *t*-tests; *p*-values < 0.05 for all replicates).

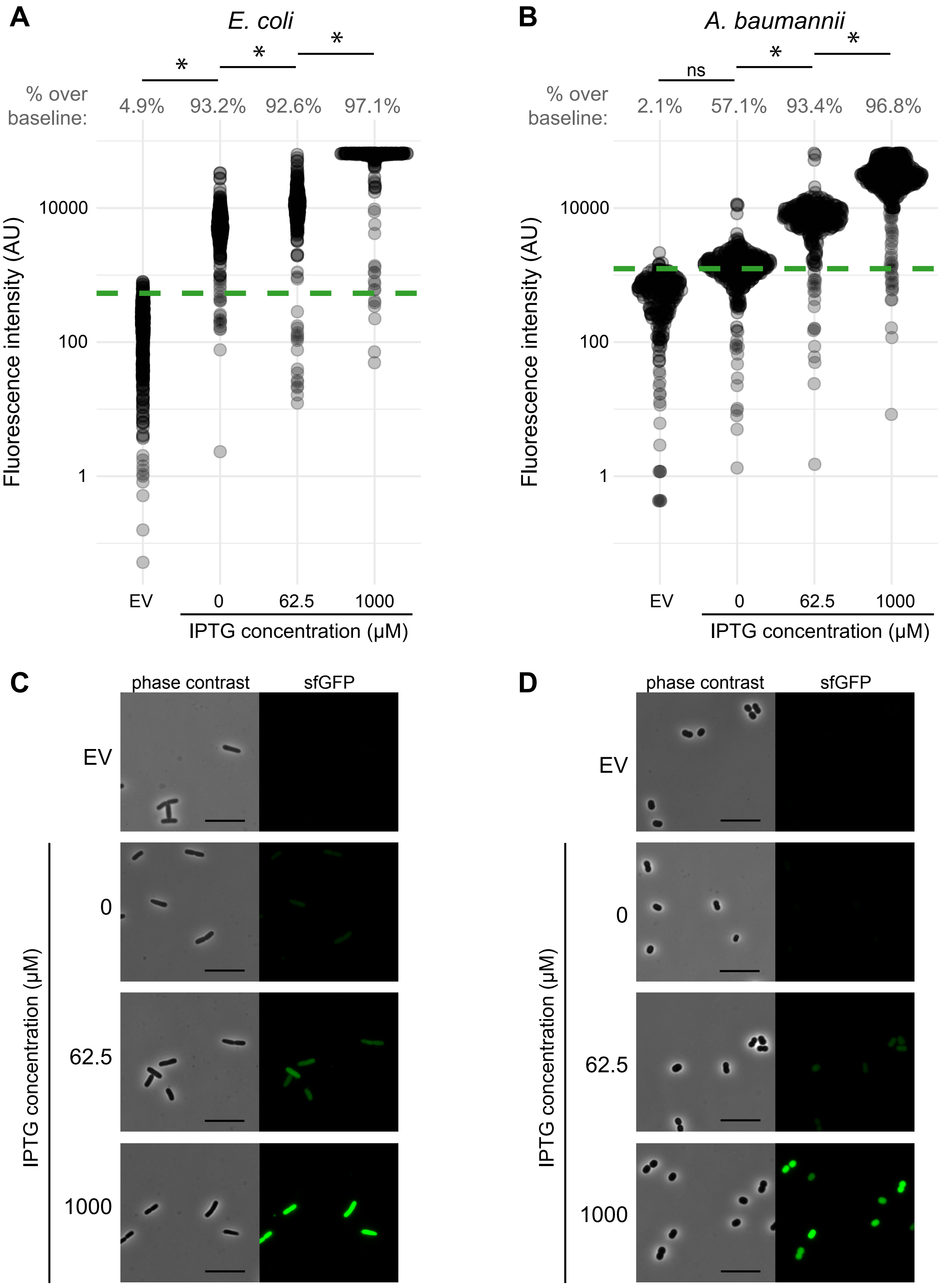
A
E. coli
*
*
*
% over baseline:
4.9%
93.2%
92.6%
97.1%
Fluorescence intensity (AU)
10000
100
1
EV
0
62.5
1000
IPTG concentration (μM)
B
A. baumannii
ns
*
*
% over baseline:
2.1%
57.1%
93.4%
96.8%
Fluorescence intensity (AU)
10000
100
1
EV
0
62.5
1000
IPTG concentration (μM)
C
phase contrast
sfGFP
EV
0
62.5
1000
IPTG concentration (μM)
D
phase contrast
sfGFP
EV
0
62.5
1000
IPTG concentration (μM)

Figure S4. Single-cell P_{abstBR} expression via microscopy.

Quantification of single-cell sfGFP fluorescence from microscopy images of **(A)** *E. coli* BW25113 or **(B)** *A. baumannii* ATCC 17978. sfGFP was expressed from the replicative vector under control of P_{abstBR}. Points represent average fluorescence intensity across cell area for individual cells (n=200-250 for *E. coli*; n=250-450 for *A. baumannii*) induced at 0, 62.5, or 1000 μM IPTG. EV are empty vector (no GFP) control samples in 1 mM IPTG. Green dotted line represents a baseline for fluorescence (empty vector mean + 1.96*standard deviation). Percent of sample measurements above baseline are reported. Asterisks and ns indicate significant and not significant sample differences, respectively (Welch's *t*-tests; *p*-values < 0.05). Representative images for **(C)** *E. coli* and **(D)** *A. baumannii* samples are shown. Ruler is 10 microns.

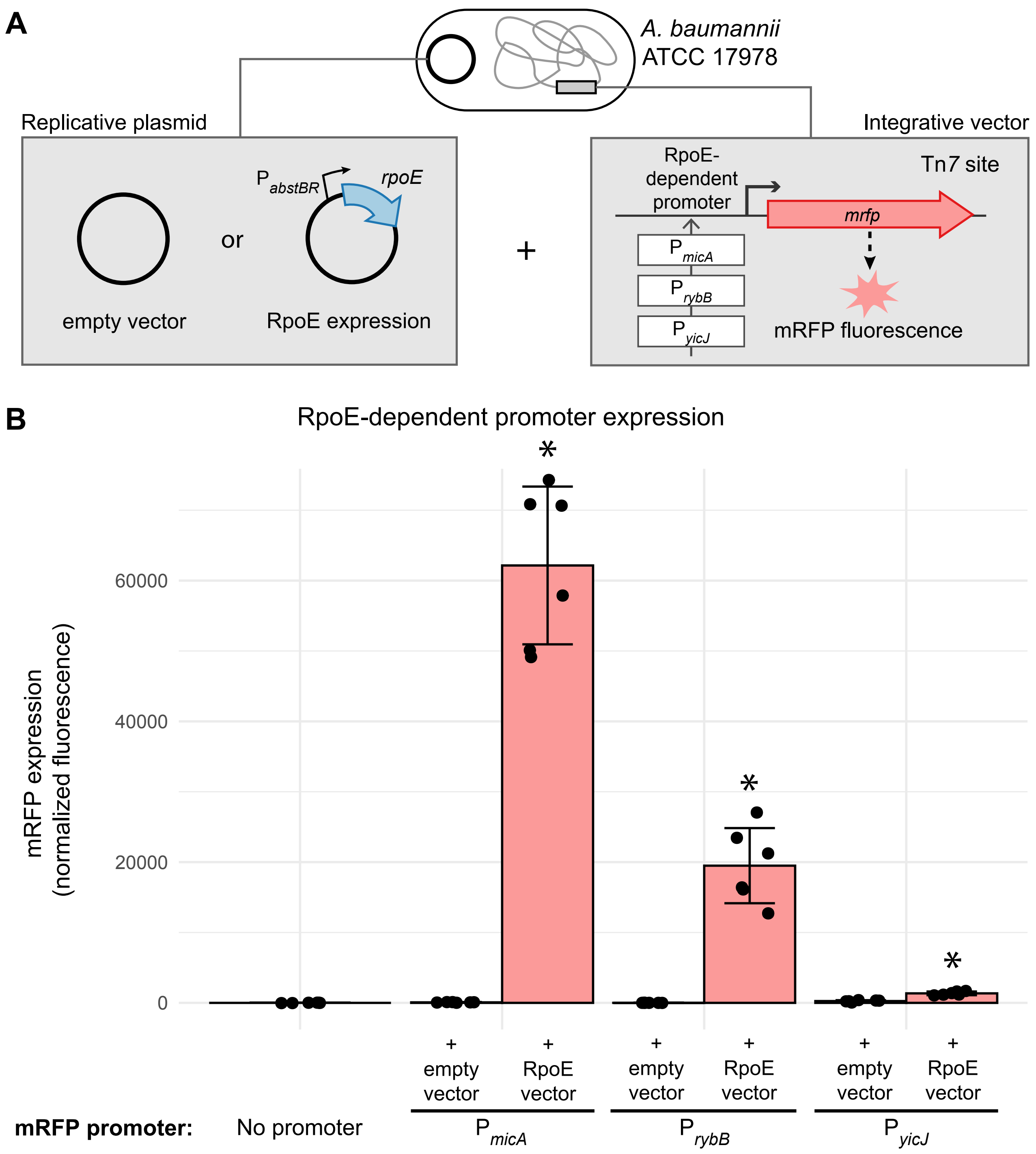

Figure S5. RpoE-dependent promoter expression in *A. baumannii*.

(A) Graphical depiction of reporter assay experiments. Strains contain a replicative plasmid (P_{abstBR}-*rpoE* overexpression vector or empty vector control) and an mRFP reporter under control of the *E. coli*-native and known RpoE-dependent *micA*, *rybB*, or *yicJ* promoters in the att_{Tn7} site (constructed using the Tn*7* integrative vector). **(B)** Bar graphs of mRFP fluorescence from promoters with and without expression of RpoE *in trans* from the replicative plasmid. Fluorescence is normalized to no promoter control, and individual data points and standard deviation are displayed (n=6 biological replicates). Asterisks indicate significant differences with RpoE expression *in trans* (Welch's *t*-tests; *p*-values < 0.05).
